# Effects of anti-CD4/CD8 antibodies in the recovery of salivary glands in experimental hyperglycemic condition

**DOI:** 10.1186/1758-5996-7-S1-A138

**Published:** 2015-11-11

**Authors:** Raphael Franco Netto, Renato Bortolatti, Fernanda Rojas, Luis Antonio Peroni, Renato Ferretti, Angel Pifferrer, Magda Jaciara Andrade, Marcos Ferreira, Eduardo Jose Caldeira

**Affiliations:** 1 Universidad Internacional Tres Fronteiras/Uninter, Ponta Porã, Brazil

## Background

Diabetes affects the metabolism, promoting damage in different tissues, including salivary organs. Current treatments, are ineffective to recovery of these tissues. In this aspect, the immunotherapy has been tested, but still remains inefficient as an agent for the control of damage caused by diabetes. In this way, more tests are necessary to conclude the real effects of this therapy.

## Objectives

Thus, the aim of this study was to evaluate the association in anti-CD4 and anti-CD8 monoclonal antibody in the recovery of salivary glands of diabetic NOD mice.

## Materials and methods

Fifteen spontaneously diabetic mice (NOD) were divided into three groups with 5 animals each: group I (Balb/C control mice), group II (untreated NOD mice), group III (NOD mice treated with anti-CD4/CD8 antibodies). The anti-CD4/CD8 antibodies (IMUNY, Rheabiotech Ltda, Brazil) were administered by intravenously injections (25 µg/days: 0, 7, 14, and 21). After treatment salivary glands samples were analyzed by light microscopy and stereology (ethical approval process: 304/11). Analysis of variance (ANOVA) and Kruskal-Wallis nonparametric test were used.

## Results

Elevated levels of glucose (mg/dL) were observed in untreated animals (group II) (605.25±31.23 p≤0.05), whereas in treated animals (group III), were noted a decrease in these levels (464.77±39.66 p≤0.05). Tissue restructure, characterized by cell volume recovery, also was observed in the group III (nuclear volume of parotid glands: 109.91±02.03 p≤0.05 and in submandibular glands: 107.52±02. p≤0.05 and cytoplasmic volume of parotid glands: 356.14±26.34 p≤0.05 and in submandibular glands: 331.22±32.11 p≤0.05; respectively).

## Conclusions

This treatment was effective in the recovery of salivary acinar cells, contributed also to homeostasis of body metabolism. Thus, this immunomodulation promoted a beneficial effect on the recovery of these tissues.

